# Reduction of Oxidative Stress in Peripheral Blood Mononuclear Cells Attenuates the Inflammatory Response of Fibroblast-like Synoviocytes in Rheumatoid Arthritis

**DOI:** 10.3390/ijms222212411

**Published:** 2021-11-17

**Authors:** Ha-Reum Lee, Su-Jin Yoo, Jinhyun Kim, Chan Keol Park, Seong Wook Kang

**Affiliations:** 1 Division of Rheumatology, Department of Internal Medicine, Chungnam National University Hospital, 282 Munhwaro, Daejeon 35015, Korea; hareum_lee@cnu.ac.kr (H.-R.L.); sujin428@cnuh.co.kr (S.-J.Y.); jkim@cnuh.co.kr (J.K.); 2 Research Institute for Medical Sciences, School of Medicine, Chungnam National University, 266 Munhwaro, Daejeon 35015, Korea; 3 Division of Rheumatology, Department of Internal Medicine, Chungnam National University Sejong Hospital, 20 Bodeum-7-ro, Sejong 30099, Korea; plutocys@cnuh.co.kr

**Keywords:** rheumatoid arthritis, regulatory T cells, helper T cells, reactive oxygen species, cytokines

## Abstract

The production and oxidation mechanism of reactive oxygen species (ROS) are out of balance in rheumatoid arthritis (RA). However, the correlation between ROS and T cell subsets in RA remains unclear. Peripheral blood mononuclear cells (PBMCs) from patients with RA (n = 40) and healthy controls (n = 10) were isolated from whole blood samples. Synovial tissues (n = 3) and synovial fluid (n = 10) were obtained from patients with RA. The repartition of T cell subsets and expression of ROS and cytokines were examined according to RA severity. Fibroblast-like synoviocytes (FLSs) from patients with RA were stimulated with PBMCs and the expression of inflammation-related molecules were measured by RT-PCR and cytokine array. Regulatory T cells from patients with moderate (5.1 > DAS28 ≥ 3.2) RA showed the highest expression of mitochondrial ROS among the groups based on disease severity. Although ROS levels steadily increased with RA severity, there was a slight decline in severe RA (DAS28 ≥ 5.1) compared with moderate RA. The expression of inflammatory cytokines in RA FLSs were significantly inhibited when FLSs were co-cultured with PBMCs treated with ROS inhibitor. These findings provide a novel approach to suppress inflammatory response of FLSs through ROS regulation in PBMCs.

## 1. Introduction

Rheumatoid arthritis (RA) is an autoimmune disease characterized by synovial inflammation, synovial lining hyperplasia, and uncontrolled autoantibody production that results in cartilage damage and bone destruction [1]. T cells make up a substantial portion of the inflammatory cells in synovial tissue and synovial fluid in patients with RA [2]. A large amount of type 1 helper T cells and IL-17–producing helper T cells (TH17s) infiltrate from peripheral blood into the synovium, where they secrete abundant pro-inflammatory cytokines/chemokines [3], induce the activation of fibroblast-like synoviocytes (FLSs) and synovial inflammation, and eventually contribute to disease severity [4]. On the other hand, regulatory T cells (Tregs) are considered an ideal therapeutic target in autoimmune diseases related to conditions such as allergy and organ transplantation [5]. It is well known that Tregs regulate the initiation and progression of RA, and that functional blockade of Tregs influences RA immunopathogenesis [6,7,8,9]. In the early stages of RA, Tregs can downregulate the effector T cell response to help maintain joint homeostasis [10]. Although Treg expansion is often detected in inflamed joints, the suppressive function of Tregs is certainly impaired in RA, and T cell homeostasis is broken [11,12,13].

Patients with RA have higher levels of reactive oxygen species (ROS) in their peripheral blood than healthy individuals [14,15]. Although proper levels of intracellular ROS play a key role in cellular function as a secondary messenger, excessive ROS levels contribute to oxidative stress-induced damage and cause local and systemic inflammatory responses [16]. ROS can directly regulate the cascade signaling of nuclear factor (NF)-κB or hypoxia-inducible factor-1α (HIF-1α), which mediate secretion of inflammatory cytokines, transmigration Cellsigration of leukocytes, hyperplasia of FLS, degradation of matrix, and joint tissue damage [17,18]. Mitochondrial ROS are closely related to specific aspects of cellular metabolism and inflammation, especially T cell receptor activation, effector T cell differentiation, and TH17/Treg balance [19,20,21]. Although ROS are closely linked to RA pathogenesis, the relation between circulating T cells and ROS remains unclear in RA.

In this study, we asked whether there is a correlation between ROS reduction in peripheral blood mononuclear cells (PBMCs) and RA pathogenesis. We first analyzed T cell subsets and their ROS levels in the peripheral blood of patients with RA. We then co-cultured FLSs with PBMCs treated with ROS inhibitor and analyzed the gene expression related to RA inflammation in the FLSs ex vivo. We found that mitochondrial ROS production in Treg cells increased according to disease severity in patients with RA. When ROS production was suppressed in PBMCs, the inflammatory response in FLSs was significantly reduced. These findings show that ROS in PBMCs might be associated with RA disease severity and contribute to RA progression.

## 2. Results

### 2.1. The Repartition of CD3^+^ T Cells Differs According to RA Severity

**Figure 1:**
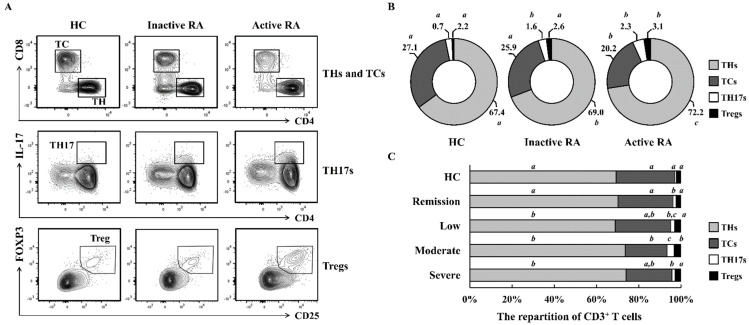
Repartition of T cell subsets in PBMCs according RA disease activity. (**A**,**B**) PBMCs were isolated from active RA (n = 20), inactive RA (n = 20), and healthy controls (n = 10; Table 1). Cells were analyzed with specific markers as described in the Supplementary Data S1. Data represent results of one median experiment in panel A and the averages of T cell subpopulations were displayed in panel B. Different letters (*a*, *b*, *c*) in panel B correspond to statistical differences of each T cell subsets among healthy control, Inactive RA, and Active RA groups (*p* < 0.05), as determined by One-way ANOVA with S-N-K’s post hoc test for multiple comparisons. (**C**) For a more detailed classification, patients in panels B were separated into four groups based on RA activity. The distribution of T cell subsets was analyzed using flow cytometry (n = 10 per group; Table 1). Statistical analysis among human subject groups was performed by one-way ANOVA, followed by Duncan’s post hoc test. Different letters (*a*, *b*, *c*) indicate statistically significant differences of each T cell subsets among healthy control, patients in the remission, low, moderate, and severe RA groups (*p* < 0.05).

We investigated whether RA severity is relevant to the characteristics of T cell subsets among PBMCs. Patients with active RA (DAS28 ≥ 3.2 and n = 20) had greater proportions of helper T cells (THs; CD3^+^CD4^+^CD8^−^), IL-17-producing helper T cells (TH17s; CD3^+^CD4^+^IL-17^+^), and Tregs (CD3^+^CD4^+^CD25^+^FOXP3^+^) and smaller proportions of cytotoxic T cells (TCs; CD3^+^CD8^+^CD4^−^) than patients with inactive RA (DAS28 < 3.2 and n = 20; Figure 1A,B). All the T cell subsets of Active RA group showed a significant difference compared with healthy controls (*p* < 0.05). To determine the relationship between RA severity and T cell subsets more −precisely, we divided these patients into four groups as described in the Materials and Methods section (n = 10 per groups). Patients with moderate RA exhibited the largest TH17 and Treg populations and the smallest TC populations among the four RA groups (Figure 1C). The frequency of TH17s was increased by 4.83-fold in the patients with moderate RA compared with that in healthy controls. The patients with moderate RA also showed higher TH17/Treg ratios than the healthy controls (3.57-fold). The populations of TH17s and Tregs steadily increased with RA activity, although they declined slightly from moderate RA to severe RA. These data indicate that repartition of CD3^+^ T cells is abnormal in patients with RA.

### 2.2. Mitochondrial ROS Levels Differ among T Cell Subsets According to RA Severity

**Figure 2:**
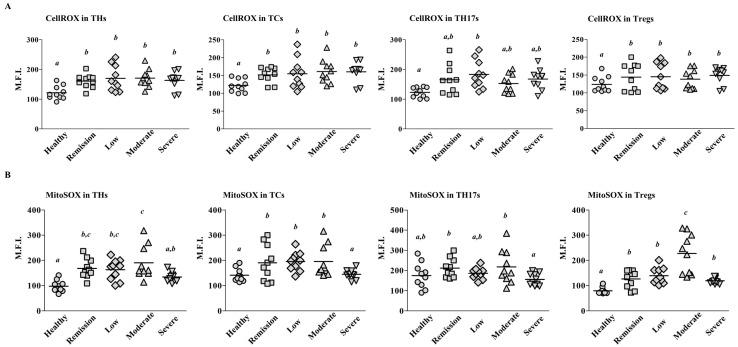
ROS expression among T cell subsets. PBMCs were isolated from patients with Severe, Moderate, Low, and Remission RA and healthy controls (n = 10 per group; Table 1). The expression levels of CellROX (**A**) or MitoSOX (**B**) in each T cell subset were measured using flow cytometry. Data are shown as the mean fluorescence intensity (M.F.I.). Each symbol represents an individual donor. The bar represents the mean. Statistical analysis among human subject groups was performed by one-way ANOVA, followed by Duncan’s post hoc test. Different letters (*a*, *b*, *c*) indicate statistically significant differences of ROS expressions among healthy control, patients in the remission, low, moderate, and severe RA groups (*p* < 0.05).

To investigate whether ROS levels are relevant to RA activity, we stained PBMCs with CellROX and MitoSOX. CellROX detects ROS in both the nucleus and mitochondria, whereas MitoSOX selectively measures mitochondrial ROS. The CellROX staining indicated that ROS levels in THs, TCs, and Tregs were higher among the patients with RA than among the controls (n = 10 per groups; Figure 2A); however, there were no differences among the four groups of patients based on RA severity. By contrast, the MitoSOX staining showed that mitochondrial ROS levels in Tregs were significantly higher in patients with Moderate RA than patients with remission, low RA, or severe RA (*p* < 0.05; Figure 2B). Because the TH population includes Tregs, the enhanced mitochondrial ROS levels in THs may reflect the elevated ROS levels in Tregs. The mitochondrial ROS levels in all the T cell subsets were lower among patients with severe RA than among patients with mderate RA, despite the trend of increasing ROS levels with increasing RA activity. Such a phenomenon is similar to the trend in Treg population sizes shown in Figure 1C. These data show that mitochondrial ROS production in Tregs increases according to RA disease activity.

### 2.3. Synovial Fluid-Derived Mononuclear Cells (SFMCs) Have Higher ROS Levels and T Cell Population Than PBMCs

**Figure 3:**
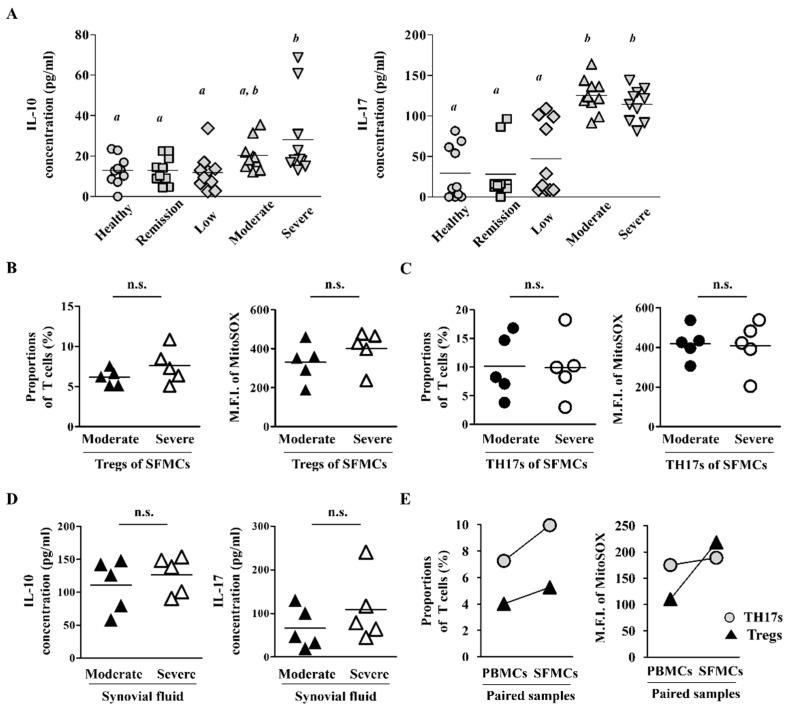
Tregs and TH17s had similar activities in moderate and severe RA. (**A**) Levels of secreted IL-10 and IL-17 were measured in sera from patients with severe, moderate, low, and remission RA and healthy controls (n = 10 per group). Each symbol represents an individual donor. Statistical analysis among human subject groups was performed by one-way ANOVA, followed by Duncan’s post hoc test. Different letters (*a*, *b*) indicate statistically significant differences of cytokine levels among healthy control, patients in the remission, low, moderate, and severe RA groups (*p* < 0.05). (**B**,**C**) Proportions of Tregs/TH17 and their expression of ROS levels were measured in SFMCs (n = 5 per group). (**D**) Levels of IL-10 and IL-17 in synovial fluid from patients with moderate and severe RA were examined (n = 5 per group). (**E**) Proportions or MitoSOX levels of Tregs/TH17 were measured in paired samples of PBMCs and SFMCs from patients with severe RA (n = 2). Each symbol corresponds to one pair from the same donor. The bars in panels (**A**–**D**) represent the mean. n.s. = not significant.

To investigate whether the difference in Treg population according to RA severity is due to transmigration of Tregs from blood into inflamed synovium tissues, we first examined the levels of IL-10 and IL-17, the representative cytokines of Tregs and TH17s, in the sera of patients with RA. The serum cytokine levels were the same between patients with severe RA and patients with moderate RA, although they were higher than the control group (n = 10 per groups; Figure 3A). In addition, the patients with severe RA seemed to maintain Treg and TH17 function close to that observed in patients with moderate RA. Next, we analyzed ROS levels in Tregs and TH17s among SFMCs and found no significant difference between patients with moderate RA and patients with severe RA (n = 5 per groups; Figure 3B,C). The secretion levels of IL-10 and IL-17 in synovial fluid were also similar between patients with Moderate RA and patients with Severe RA (n = 5 per groups; Figure 3D). When we compared paired samples of PBMCs and SFMCs from patients with severe RA, the T cell proportions and ROS expression were higher in SFMCs than in PBMCs (n = 2; Figure 3E). Taken together, these results suggest that Tregs and TH17s in the peripheral blood of patients with severe RA may accumulate in inflamed joint tissues.

### 2.4. Inflammatory Response of FLS Is Suppressed via ROS Inhibition in PBMCs

**Figure 4:**
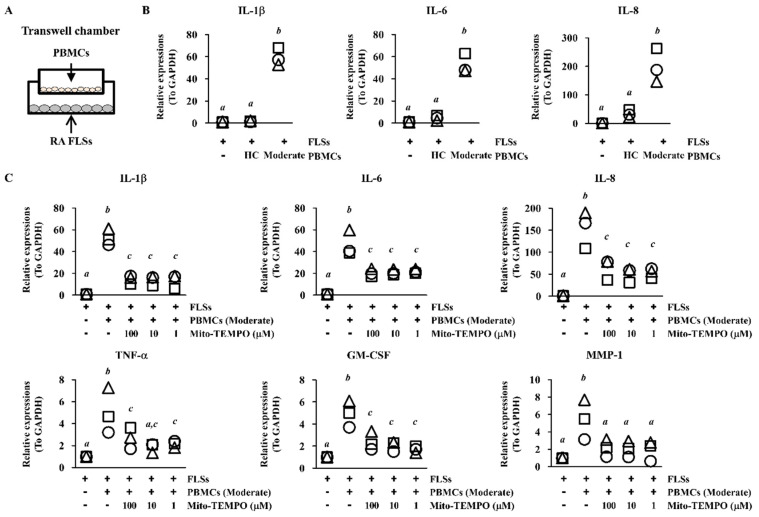
Mito-TEMPO reduced the pro-inflammatory effects of PBMCs on FLSs. (**A**) Wells with transwell inserts were seeded with FLSs in the lower chamber and PBMCs in the upper chamber. The combined transwell chambers were incubated for 24 h, and FLSs were analyzed for expression of inflammatory genes using RT-PCR (**B**) Total mRNA was extracted from FLSs, and expression of *IL-1β*, *IL-6*, and *IL-8* was assessed (n = 3 per group). Each symbol represents an individual donor. (**C**) Upper chambers were loaded with PBMCs that had been pre-incubated with 1 μM, 10 μM, or 100 μM Mito-TEMPO for 30 min and then washed with PBS. The PBMCs were incubated with FLSs for 24 h. Expression of *IL-1β*, *IL-6*, *IL-8*, *TNF-α*, *GM-CSF*, and *MMP-1* was assessed in the FLSs by RT-PCR. *GAPDH* was used as a control. Data represent three individual samples from patients with moderate RA activity. Each symbol represents an individual donor. Bars represent the mean. Statistical analysis was performed by one-way ANOVA, followed by Duncan’s post hoc test. Different letters (*a*, *b*, *c*) indicate statistically significant differences of cytokine expressions among sample groups (*p* < 0.05).

**Figure 5:**
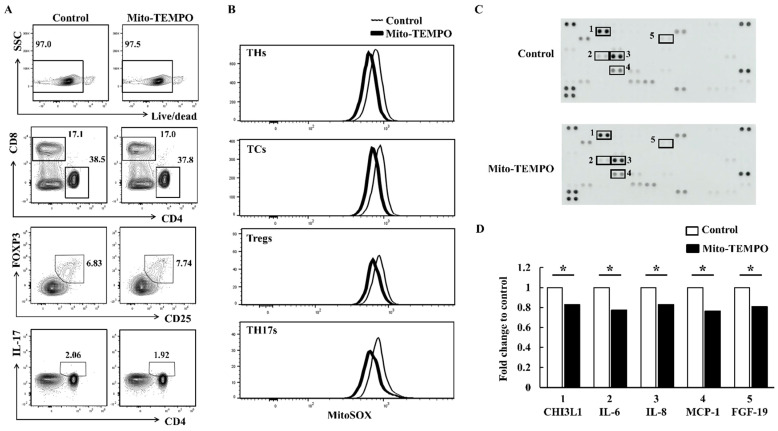
T cell populations and ROS expression in Mito-TEMPO–treated PBMCs. (**A**,**B**) PBMCs were isolated from patients with moderate RA activity and incubated with 10 μM Mito-TEMPO for 30 min. The PBMCs were then indirectly incubated with FLSs using transwell inserts for 24 h. T cell populations and mitochondrial ROS expression were then analyzed in the PBMCs. Control cells are represented by the black thin line, and Mito-TEMPO–treated PBMCs are indicated by the bold line. Data represent results of one median experiment. Data represent results of one experiment, which was performed in triplicate with similar results. (**C**,**D**) PBMCs from patients with moderate RA were pretreated with 10 μM Mito-TEMPO for 30 min and then incubated with FLSs using transwell inserts. After 24 h, the supernatants of the FLSs were analyzed using a cytokine array according to the manufacturer’s instructions. The array images are shown in (**C**). 1: Chitinase-3-like protein 1 (CHI3L1); 2: IL-6; 3: IL-8; 4: monocyte chemoattractant protein-1 (MCP-1); 5: fibroblast growth factor 19 (FGF-19). Dot density represents the fold change relative to the control (**D**). White bars indicate controls, and black bars indicate the supernatants of FLSs co-cultured with 10 μM Mito-TEMPO–treated PBMCs. Data represent results of one median experiment in panel C and the averages of cytokine secretion were displayed in panel D (n = 3). Statistical analysis was performed using the paired Student’s *t*-test. * indicates *p* < 0.05.

FLS activation leads to degenerative bone degradation through the production of inflammatory cytokines/chemokines [22]. When we incubated PBMCs from patients with RA indirectly with FLSs using transwell inserts, the expression of IL-1β, IL-6, and IL-8 was higher in FLSs exposed to PBMCs from patients with moderate RA than in FLSs exposed to PBMCs from healthy controls (n = 3 per groups; *p* < 0.05; Figure 4A,B). To investigate whether suppression of ROS levels affects joint inflammation, we pre-incubated PBMCs from patients with moderate RA with Mito-TEMPO, a mitochondria-specific ROS inhibitor. We then co-incubated the PBMCs with FLSs for 24 h using transwell inserts. FLSs co-cultured with Mito-TEMPO–treated PBMCs had significantly reduced mRNA levels of IL-1β, IL-6, IL-8, tumor necrosis factor (TNF)-α, granulocyte-macrophage colony-stimulating factor (GM-CSF), and matrix metallopeptidase (MMP)-1 than FLSs co-cultured with untreated PBMCs (n = 3 per groups; *p* < 0.05; Figure 4C). Among the T cell subsets, Treg proportions were slightly increased by the Mito-TEMPO treatment, whereas the other T cell subsets were unaffected (Figure 5A). We detected ROS suppression in all the T cell subsets from FLSs incubated with PBMCs from patients with moderate RA (Figure 5B). When we examined FLSs stimulated with Mito-TEMPO–treated PBMCs using a cytokine array, the inflammatory protein levels in the stimulated FLSs were generally lower than those in control FLSs incubated with untreated PBMCs (n = 3 per groups; *p* < 0.05; Figure 5C,D). These data show that mitochondrial ROS in PBMCs may induce RA progression via FLS activation.

## 3. Discussion

Several studies have investigated whether abnormal repartition of T cell subsets is associated with the pathogenic mechanisms of RA [23]. Previous studies showed that the proportion of CD25^+^Foxp3^+^ Tregs in peripheral blood did not differ between patients with RA and control subjects [24,25], whereas other groups reported that the percentage of circulating CD4^+^CD25^high^ Tregs in patients with RA was significantly elevated than that in controls [26,27]. In this study, we showed that the frequencies of Tregs and TH17s were increased in the peripheral blood of patients with RA compared with those in the peripheral blood of controls. Tregs with low FOXP3 expression have a non-suppressive function, and an increase in the proportion of resting Tregs can cause progression of RA disease [28]. Accordingly, when the proportion of resting Tregs with dysfunctional suppressive activity was increased, the immune-suppressive activity of Tregs was insufficient to suppress RA development [29]. Similarly, in the autoimmune disease systemic lupus erythematosus, increases in the proportion of resting Tregs and impairment of the suppressive function of Tregs were correlated with clinical disease activity [30]. FOXP3-expressing Tregs have a different immune-suppressive activity, however, and further study is needed to analyze the suppressive function of Tregs in RA.

All RA groups in our study showed higher intracellular ROS production than healthy controls (Figure 2A). These data confirmed that patients with RA had more ROS generation in circulating T cells than healthy controls. Mitochondrial ROS levels in Tregs were much more dependent on RA activity than nuclear ROS levels in Tregs, but they were slightly lower in patients with Severe RA than in patients with moderate RA (Figure 2B). When T cell populations and ROS expression were analyzed in paired samples of SFMCs and PBMCs from patients with severe RA, SFMCs had higher proportions of Tregs and TH17s and higher mitochondrial ROS levels than PBMCs (Figure 3E). Therefore, large numbers of Tregs may transmigrate from peripheral blood to inflamed synovium in patients with severe RA (Figure 3E). Although it is well known that T cells levels are higher in RA synovium than in peripheral blood, further study of the migratory ability of circulating Tregs is required [27]. On the other hand, it was reported that Treg numbers increased after patients with RA were treated with a disease-modifying antirheumatic drug (DMARD) [31]. In the present study, half of severe RA patients have not taken any DMARDs (Table 1). Therefore, it is possible that the decrease of Treg cells in severe RA compared to moderate RA can be ascribed to the difference in the rate of receiving DMARDs.

The RA synovium typically displays abnormal glycolysis and mitochondrial dysfunction, corresponding to accumulation of excessive lactic acid and ROS [32]. Although proper ROS levels are essential for cell function, immoderate ROS levels lead to DNA damage, cellular aging, and oxidative stress-mediated signal activation [33]. FLSs incubated indirectly with PBMCs from patients with RA expressed more IL-1β, IL-6, and IL-8 mRNAs than FLSs incubated with PBMCs from healthy controls or FLSs alone (Figure 4B). Reduced levels of ROS in PBMCs from patients with RA significantly attenuated the inflammatory response of FLSs (Figure 4C and Figure 5C,D). When we treated PBMCs with the widely used antioxidant N-acetylcysteine (NAC), the inflammatory response of FLS was similarly inhibited (data not shown). Because Tregs had the highest expression of mitochondrial ROS among the T cell subsets in patients with moderate RA activity (Figure 2B), the strongest effect of Mito-TEMPO may be on Tregs. These results suggest that the high level of ROS could weaken the suppressive function of Tregs and lead to higher prevalence and more severe symptoms of RA. A previous study found that intracellular ROS levels are upregulated in senescent Tregs and lead to impaired suppressive function and uncontrolled inflammation [34]. They revealed that DDB1- and CUL4-associated factor 1 (DCAF1) was downregulated in aged Tregs and was critical to restrain Treg aging via ROS regulated by glutathione-S-transferase P (GSTP1). This means that ROS axis controlled directly Treg suppressive function, because treatment with the ROS scavenger NAC restored the proliferation and immune-suppressive function of senescent Tregs. On the other hand, Tregs in autoimmune disease are associated with senescent phenotypes and impaired suppressive [35]. Fessler et al. have suggested that CD4^+^FOXP3^+^CD28^−^ T cells might be a novel type of senescent Treg with reduced suppressive function, increased production of pro- and anti-inflammatory cytokines, and a positive correlation with clinical parameters of RA [36]. When we compared the expression of aging biomarker p16 (INK4a), their levels in Tregs were higher in patients with moderate RA than healthy control (data not shown). Furthermore, PBMCs from patients with moderate RA treated with Mito-TEMPO showed significantly decreased p16 expression in Tregs (data not shown). These correlations support the hypothesis that excessive ROS levels in RA damage the suppressive function of Tregs and induce inflammatory response in joint tissues.

In conclusion, our results revealed that patients with moderate RA had the most TH17s and Tregs and the least TCs among RA groups. The elevated mitochondrial ROS levels in circulating Tregs are correlated with RA disease severity. In addition, suppression of ROS in PBMCs from patients with RA significantly inhibited the inflammatory response of FLSs. Further studies are needed to determine whether the mitochondrial dysfunction is specific to certain Treg subpopulations, and whether the dysfunction is directly involved in RA activity. Further studies of mitochondrial ROS in Tregs may contribute to a better understanding of RA progress and development and support the development of novel therapeutic strategies for RA disease.

## 4. Materials and Methods

### 4.1. Human Subjects

Blood samples were collected from patients with RA (n = 40) and healthy adult volunteers (n = 10) at Chungnam National University Hospital (Daejeon, Korea). Patients were diagnosed with RA according to American College of Rheumatology (ACR)/European League Against Rheumatism (EULAR) 2010 classification criteria [37]. The RA activity in the patients was categorized according to the Disease Activity Score 28 (DAS28): severe (DAS28 ≥ 5.1), moderate (5.1 > DAS28 ≥ 3.2), low (3.2 > DAS28 ≥ 2.6), and remission (DAS28 < 2.6; n = 10 per group) [38]. Synovial tissues were obtained from patients with RA who underwent synovectomy or joint replacement (n = 3). All synovectomies were performed only for medical indications. After fat and fibrous tissues were removed, the synovium was cut into small pieces and incubated with 0.1% collagenase (Sigma-Aldrich, St. Louis, MO, USA) in Dulbecco’s modified Eagle’s medium (DMEM; Gibco, Waltham, MA, USA) at 37 °C for 3 h. The dissociated cells were then cultured in DMEM supplemented with 10% fetal bovine serum (FBS) and maintained in a 5% CO_2_ incubator at 37 °C. FLSs were used for experiments after four to six passages (n = 3).

### 4.2. Ethics Statement

This study was performed according to the recommendations of the Declaration of Helsinki and approved by the Institutional Review Board of Chungnam National University Hospital (CNUH 2015-10-052). All the study patients signed an informed written consent before participation.

### 4.3. Isolation of PBMCs and SFMCs

PBMCs were obtained from whole blood with RA (n = 40) and healthy adult volunteers (n = 10). SFMCs were obtained from joint fluid of patients with Moderate (n = 5) and Severe (n = 5) RA using lymphocyte separation medium (Corning Inc., Corning, NY, USA) and density gradient centrifugation. Both cell types were frozen in dimethyl sulfoxide (DMSO) containing CELLBANKER1 (Zenoaq, Tokyo, Japan) and stored in liquefied nitrogen. After thawing, the cells were maintained in Roswell Park Memorial Institute culture medium (RPMI; Gibco) supplemented with 2 mM L-glutamate and 10% FBS (Gibco) at 37 °C in 5% CO_2_.

### 4.4. Flow Cytometry Analysis

To separate live and dead cell populations, PBMCs were stained with Live/Dead fixable stain dye (Life Technologies, Carlsbad, CA, USA). Then, the cells were washed in PBS and incubated with PerCP-Cy5.5-conjugated anti-CD3 (eBioscience, San Diego, CA, USA), PE-Cy7-conjugated anti-CD4 (BD Biosciences, Franklin Lakes, NJ, USA), APC-Cy7-conjugated anti-CD8 (BD Biosciences), and V450-conjugated anti-CD25 (BD Biosciences). Following fixation and permeabilization using the FOXP3/Transcription Factor Staining Buffer Set (eBioscience), the cells were stained with Alexa647-conjugated anti-FOXP3 (BD Biosciences) and Alexa488-conjugated-IL-17A (BD Biosciences) or PE-conjugated-IL-17A (BD Biosciences). CellROX Green Oxidative Stress Reagents (Molecular Probes, Eugene, OR, USA) or MitoSOX Red Mitochondrial Superoxide Indicator (Invitrogen, Carlsbad, CA, USA) were used for ROS detection. The cells were analyzed with a FACSCantoII flow cytometer (BD Biosciences), and data were processed with FlowJo software (Tree Star, OR, USA).

### 4.5. Enzyme-Linked Immunosorbent Assay (ELISA)

IL-10 and IL-17 concentrations were measured using ELISA kits for human IL-10 (BD Biosciences) or human IL-17 (R&D Systems, Minneapolis, MN, USA) according to the manufacturers’ instructions. Levels were estimated by interpolation from a standard curve generated using a Sunrise absorbance reader (Tecan, Männedorf, Switzerland) at 450 nm.

### 4.6. Co-Culture Using Transwell Inserts and Cytokine Array

FLSs were pre-seeded on the bottom chamber of a transwell plate for 24 h. PBMCs were treated with Mito-TEMPO (Sigma-Aldrich) for 30 min, washed in PBS, and then loaded onto 0.4 μm pore transwell inserts (Corning Inc., Corning, NY, USA). The transwell chambers were incubated at 37 °C for 24 h, after which the FLSs were examined to determine mRNA levels of inflammation-related genes. The culture supernatants of FLSs were used for cytokine profiling with the Proteome Profiler array (cat# ARY022B and R&D Systems) according to the manufacturer’s instructions. Quantification of cytokine optical densities was obtained with an Amersham Imager 680 (GE Healthcare, Chicago, IL, USA) and analyzed using the Quick Spots Tool (Western Vision, Salt Lake, UT, USA). The PBMC:FLS ratio in all experiments was 10:1.

### 4.7. Real-Time PCR

Total RNA was extracted using TRI Reagent (Molecular Research Center, Cincinnati, OH, USA) according to the manufacturer’s instructions. The RNA was used in reverse-transcription reactions with ReverTra Ace^®^ qPCR RT Master Mix (TOYOBO, Osaka, Japan) according to the manufacturer’s instructions. SYBR^®^ Green Realtime PCR Master Mix (TOYOBO) was used for real-time PCR analysis of cDNA according to the manufacturer’s instructions. The primers were synthesized by Bioneer (see Table 2 for primer sequences). Thermal cycling conditions were as follows: initial denaturation at 95 °C for 5 min, followed by 40 cycles of 95 °C for 10 s, 60 °C for 15 s, and 72 °C for 20 s. A melting step was performed by raising the temperature from 72 °C to 95 °C after the last cycle. Thermal cycling was conducted on a CFX Connect Real-Time PCR Detection System (Bio-Rad Laboratories, Hercules, CA, USA). The target-gene expression levels are shown as a ratio in comparison with the level of glyceraldehyde 3-phosphate dehydrogenase (GAPDH) in the same sample according to the cycle threshold (Ct) value. The relative expression levels of target genes were calculated by the 2^−ΔΔCT^ relative quantification method.

### 4.8. Statistical Analysis

Results are presented as means. The level of significance, determined at the 95% confidence limit or greater (*p* < 0.05), was calculated by one-way analysis of variance (ANOVA) followed by S-N-K’s post hoc test or Duncan’s post hoc test using SPSS 22.0 (IBM, Armonk, NY, USA). Different letters (a, b, c) indicate statistically significant differences between groups (*p* < 0.05).

## Figures and Tables

**Table 1:** Clinical and laboratory characteristics of human subjects. Values are number (%) or mean ± SD (Range). Kruskal-Wallis test was used for Sex, Onset age, Rheumatoid factor (+), Rheumatoid factor titer, ACPA (+), and Steroid analyses. One way ANOVA test was applied for analyses of Age and DAS28. Mann-Whitney test was used for treatment time, Duration of disease, ACPA titer, SJC, TJC, VAS, ESR, CRP, Duration of treatment, and naïve analyses. In *p*-value analysis, 1: Severe, 2: Moderate, 3: Low, 4: Remission patients with RA. SD: standard deviation; ACPA: Anti-cyclic-citrullinated protein; SJC: swollen joint count; TJC: tender joint count; VAS: visual analogue scale; ESR: erythrocyte sedimentation rate: CRP: C-reactive protein; DMARD: Disease-modifying anti-rheumatic drug.

		Group	Active RA (DAS28 ≥ 3.2)		Inactive RA (DAS28 < 3.2)		Health (n = 10)	*p*-Value
Characteristics			Severe (n = 10)	Moderate (n = 10)	Low (n = 10)	Remission (n = 10)		
Female, n (%)			7 (70)	7 (70)	8 (80)	7 (70)	7 (70)	0.983
Age, years			56.9 ± 7.5 (44–68)	55.2 ± 14.0 (30–72)	59.4 ± 12.8 (35–80)	53.0 ± 12.8 (28–69)	56.1 ± 12.0 (35–70)	0.823
Onset age, years			55.8 ± 6.4 (44–64)	51.2 ± 14.3 (21–68)	53.5 ± 15.2 (24–67)	47.0 ± 14.3 (28–69)		0.484
From onset time to treatment time, months			14.4 ± 29.1	13.6 ± 18.86	71.2 ± 76.82	33.5 ± 46.89		2,3:0.023
Duration of disease, months			27.8 ± 42.9 (2–120)	65.0 ± 56.5 (1.5–151)	133.4 ± 87.8 (18–276)	98.4 ± 70.0 (14–228)		1,4:0.003 1,3:<0.001 2,3:0.035
Rheumatoid factor–positive, n (%)			9 (90)	9 (90)	8/9 (89)	7/9 (78)		0.603
Rheumatoid factor titer (IU/mL)			94.1 ± 69.4	106.2 ± 81.0	145.2 ± 119.6	92.6 ± 88.4		0.743
Anti CCP antibody–positive, n (%)			10 (100)	6/7 (86)	5/5 (100)	5/6 (83)		0.489
Anti CCP antibody titer (U/mL)			367.2 ± 174.8	172.6 ± 181.2	500 ± 0	189.3 ± 243.7		1,2:0.019 2,3:0.003
DAS28 (ESR)			6.3 ± 0.8	4.2 ± 0.5	2.8 ± 0.3	1.4 ± 0.6		<0.001
SJC			11.2 ± 7.5	1.6 ± 1.6	0.1 ± 0.3	0.1 ± 0.3		1,2 <0.001 1,3 <0.001 1,4 <0.001 2,3:0.004 2,4:0.004
TJC			11.5 ± 7.1	1.8 ± 1.6	0.4 ± 0.7	0.1 ± 0.3		1,2 <0.001 1,3 <0.001 1,4 <0.001 2,4 <0.001
VAS			73 ± 15.0	41 ± 15.2	16.5 ± 9.4	9.8 ± 4.1		1,2:0.001 1,3 <0.001 1,4 <0.001 2,3 <0.001 2,4 <0.001
ESR			52.4 ± 38.3	49.5 ± 25.9	29.2 ± 17.5	8.6 ± 9.2		1,4 <0.001 2,3:0.011 2,4 <0.001 3,4:0.011
CRP			2.1 ± 2.3	1.9 ± 2.2	0.5 ± 0.8	0.06 ± 0.1		1,4:0.015 2,4 <0.001
Duration of treatment, months			2.2 ± 3.4 (0–8)	44.5 ± 54.8 (0.5–139)	79.2 ± 71.2 (6–204)	65.5 ± 59.7 (1.5–187)		1,4:0.001 1,3:<0.001 1,2:0.005 2,3:0.023
Treatment	Naïve, n (%)		5 (50)	2 (20)	0	0		0.280
	Steroid, n (%)		5 (50)	7 (70)	9 (90)	8 (80)		0.539
	Prednisolone dose (mg/day)		6.5 ± 1.3	5.5 ± 2.6	2.6 ± 1.9	4.1 ± 3.7		
	DMARD, biologic, n (%)		0	0	0	2 (20)		
	DMARD, conventional, n (%)	total	5 (50)	8 (80)	9 (90)	7 (70)		
		Methotrexate, n (%)	5 (50)	8 (80)	9 (90)	7 (70)		
		Methotrexate dose (mg /week)	11.3 ± 3.6	12.8 ± 2.8	10.8 ± 4.9	9.5 ± 6.7		
		Sulfasalazine, n (%)	3 (30)	2 (20)	3 (30)	3 (30)		
		Hydroxychloroquine, n (%)	2 (20)	6 (60)	4 (40)	3 (30)		
		Leflunomide, n (%)	0	0	3 (30)	4 (40)		

**Table 2:** Primers used for PCR.

	Sense Primer	Antisense Primer
*IL-1β*	GGATATGGAGCAACAAGTGG	ATGTACCAGTTGGGGAACTG
*IL-6*	AACCTGAACCTTCCAAAGATGG	TCTGGCTTGTTCCTCACTACT
*IL-8*	CATACTCCAAACCTTTCCACCCC	TCAGCCCTCTTCAAAAACTTCTCCA
*TNF-α*	CCCGAGTGACAAGCCTGTAG	GATGGCAGAGAGGAGGTTGAC
*GM-CSF*	TCTCAGAAATGTTTGACCTCCA	GCCCTTGAGCTTGGTGAG
*MMP-1*	GGCCCACAAACCCCAAAAG	ATCTCTGTCGGCAAATTCGTAAGC
*GAPDH*	CACATGGCCTCCAAGGAGTAA	TGAGGGTCTCTCTCTTCCTCTTGT

## Supplementary Materials

The following are available online at https://www.mdpi.com/article/10.3390/ijms222212411/s1.
