# Supplementary material for: Reduction of Oxidative Stress in Peripheral Blood Mononuclear Cells Attenuates the Inflammatory Response of Fibroblast-like Synoviocytes in Rheumatoid Arthritis
Source: Int J Mol Sci. 2021 Nov 17;22(22):12411. doi: 10.3390/ijms222212411 (PMC8624216; doi:10.3390/ijms222212411)
Supplement: Supplementary file 1 [file ijms-22-12411-s001.zip › ijms-1428747-supplementary.pdf]

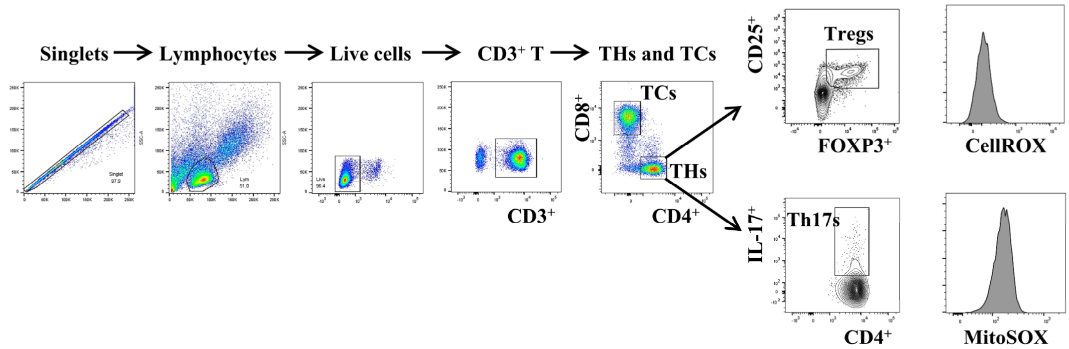

Supplementary Data S1. Gating strategy for defining T cell subpopulations and ROS expression

PBMC from patients with RA (n = 40) and healthy controls (n = 10) were isolated from whole blood samples. Single cells were gated in isolated PBMCs using FSC-A and FSC-H, and lymphocyte populations were determined with Live/Dead Aqua staining. CD3-expressing T cells were gated from live singlet lymphocytes. Following CD4 and CD8 expression, CD3$^+$ T cells were defined as THs (CD3$^+$CD4$^+$CD8$^-$) and TCs (CD3$^+$CD8$^+$CD4$^-$). Following fixation and permeabilization using FOXP3/transcription factor staining buffer, cells were stained with specific antibodies for FOXP3 and IL-17A. THs were further classified as TH17s (CD3$^+$CD4$^+$IL-17$^+$) and Tregs (CD3$^+$CD4$^+$CD25$^+$FOXP3$^+$). After washing, each T cell subset was incubated with two types of ROS dye, CellROX or MitoSOX. Plots and histograms are representative examples with PBMCs from patient with Moderate RA.
